# Developing Zika vaccines: the lessons for disease X

**DOI:** 10.1186/s13073-018-0561-2

**Published:** 2018-06-26

**Authors:** Alan D. T. Barrett

**Affiliations:** Sealy Institute for Vaccine Sciences and Department of Pathology, University of Texas Medical Branch, Galveston, TX 77555-0436 USA

## Abstract

There is an urgent need to develop vaccines against emerging diseases, including those caused by pathogens that are currently unknown to cause human disease, termed ‘disease X’. Here, Zika virus infection is considered as an example of disease X. The speed of Zika vaccine development provides optimism for our ability to prepare vaccines against unknown pathogens.

## Emerging infectious diseases

The development of vaccines for newly emerging infectious disease, especially for those caused by unknown pathogens, is an important area of public health due to the difficulties in responding rapidly to such diseases once an outbreak is established. Over 70% of emerging diseases are zoonotic and exist in animal reservoirs and/or are transmitted by insect vectors, making control nearly impossible without vaccination. Furthermore, difficulty anticipating the consequences of such an outbreak results not only in public health problems but also in financial, infrastructural, and governmental issues involved in response. The World Health Organization has developed an annually updated *Research and Development (R&D) Blueprint for Action to Prevent Epidemics* [1]. The 2018 list [2] included ‘disease X’ for the first time, which “represents the knowledge that a serious international epidemic could be caused by a pathogen currently unknown to cause human disease, and so the R&D Blueprint explicitly seeks to enable cross-cutting R&D preparedness that is also relevant for an unknown ‘disease X’ as far as possible.”

Next generation sequencing (NGS) technologies have revolutionized our ability to study not only the genomes of distinct species but also those of populations of organisms, such as in microbiome and virome projects. NGS has allowed a quantum leap in our understanding of the genomes of emerging pathogens and of the genetic variation within these genomes. Studies to date suggest that that all potential genetic groups of pathogens have been classified and no new genetic groups will be identified; however, interpretations of sequence differences and mutations are not easily equated to biological and ecological characteristics of organisms, as exemplified by the annual genetic changes in influenza viruses. The implication of NGS data is that emerging pathogens will be members of genetic groups that have already been identified, and thus that comparison of an emerging disease X pathogen with known genetically related organisms would accelerate vaccine development. Here, I discuss Zika virus (ZIKV) as an example of a pathogen causing an emerging disease X.

## Zika as a disease X

Like dengue (DEN), Japanese encephalitis (JE), West Nile, and yellow fever (YF), ZIKV is a mosquito-borne flavivirus. It was first isolated from a sentinel Rhesus macaque in the Zika forest of Uganda in 1947. Only 14 clinical cases were reported from 1951 to 2006, and these were limited in severity to an acute febrile illness (characterized by rash, conjunctival infection, arthralgia, myalgia, and headache) known as Zika fever. Not surprisingly, ZIKV was not considered to be an important human pathogen, nor to have epidemic potential. Consequently, the outbreak of Zika fever that occurred in the Federated States of Micronesia (Yap Island) in 2007 was astonishing. In 2013–2014, a large epidemic occurred in French Polynesia, which spread throughout the Pacific to New Caledonia, Vanuatu, the Cook Islands, and the Solomon Islands. A further shock came in 2015–2016 when ZIKV spread throughout the Americas, with transmission documented in more than 70 countries and territories [3]. Although 75–80% of ZIKV infections are asymptomatic, the outbreaks since 2007 have been associated with increased rates of autoimmune neurologic disorders, such as acute disseminated encephalomyelitis and Guillain-Barre syndrome. In addition, virus transmission from mother to fetus during pregnancy has manifested in congenital Zika syndrome (CZS), characterized by placental insufficiency, fetal growth restriction, oligohydramnios, ocular disorders, auditory impairments, congenital microcephaly, ventricular calcifications, migration defects, simplified gyral patterns, and cerebellar hypoplasia [4]. This supports Zika as a ‘disease X’: a pathogen that was not considered to be of public health importance mediating a range of clinical syndromes that was completely unexpected based on its history.

## Molecular biology and pathogenesis of ZIKV

Extraordinary progress has been made on understanding the molecular biology and pathogenesis of ZIKV, with PubMed listing over 4400 publications since January 2015. Much of the rapid progress has been accelerated by leveraging previous work on other flaviviruses, in particular the advances made in understanding the structure–function relationship and genomics of dengue viruses in the past 25 years. The ZIKV genome is indeed a typical flavivirus genome. A positive-sense, single-stranded RNA genome of about 10,000–11,000 nucleotides in length, it consists of 5′ and 3′ noncoding regions (NCR) and a single open reading frame encoding a polyprotein that is co- and post-translationally processed to generate three structural (capsid (C), precursor of membrane (prM) and envelope (E)), and seven nonstructural (NS) proteins (NS1–NS5) in the gene order: 5′-C-prM-E-NS1-NS2A-NS2B-NS3-NS4A-NS4B-NS5–3′ [3].

Genetic analyses have revealed two major ZIKV lineages: African and Asian. The recent American strains form a sublineage of the Asian lineage. ZIKV strains are a single serotype, and studies with polyclonal antisera and monoclonal antibodies have shown that, based on neutralization, ZIKV did not share extensive crossreactivity or antigenic determinants of neutralization with other flaviviruses. This has been critical for ZIKV vaccine development as it implies that any ZIKV strain could be used for vaccine development, that the neutralizing epitopes are analogous to those of other flaviviruses, and that there is no need for a multivalent vaccine, such as that needed with DEN [3]. Recent studies have aimed to identify the molecular determinants of the clinical pathology of ZIKV (e.g., [4]), but these results require careful interpretation as they are mostly elucidated in mouse models which may not be indicative of pathology in humans.

## Zika vaccine development

Experiences with licensed vaccines for flaviviruses, including formalin-purified inactivated vaccines (PIVs) for JE and live attenuated vaccines (LAVs) for JE, YF, and DEN, have facilitated Zika vaccine efforts. Much of Zika vaccine development has focused on utilizing prM/E in various platforms, although candidate LAVs have also used mutagenesis of infectious clones (e.g., mutation of E, NS1, or 3′-NCR) [5]. Of the vaccine candidates currently in clinical evaluation, four are ‘classical’ PIVs similar to the inactivated JE vaccine (Ixiaro™) [6], three are DNA [7, 8], one is mRNA [9], and one is measles virus-vectored [10]. The DNA and RNA vaccines utilize prM/E genes from different ZIKV strains.

**Fig. 1:**
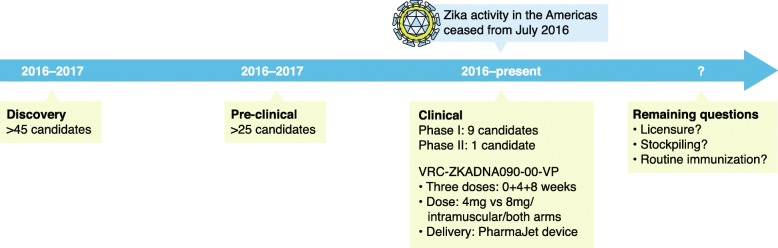
Zika vaccine development pathway. The vaccine development pathway starts with basic science/discovery and a lead candidate vaccine undergoes preclinical evaluation for safety and immunogenicity in animal models where high-quality data are needed to justify to a regulatory agency (e.g., US Food and Drug Administration (FDA) or European Medicines Agency (EMA)) that a vaccine candidate is suitable to be evaluated in clinical trials. Following successful clinical trials, a vaccine will be licensed for use

Discovery studies for Zika vaccines began in 2015, followed by preclinical advances published from mid-2016. The first phase I clinical trial results were published in late 2017 [6–8] (see Fig. 1), and a candidate DNA vaccine is currently in phase II clinical evaluation [7].

All the vaccine candidates have performed well in mice and nonhuman primates (NHPs), and a neutralization titer of approximately 1 in 100 could prevent viremia in ZIKV-challenged animals immunized with candidate vaccines [6–10]. This compares with neutralization titers of 1 in 10 for other licensed flavivirus vaccines, indicating that greater quantities of anti-ZIKV antibodies are required for protection, at least in animal models. Only the live attenuated [5] and RNA [9] ZIKV vaccine candidates induced sterilizing immunity in mice (but not in NHPs), requiring a neutralization titer of around 1 in 5000. Recent studies have shown that PIV and an adenovirus-vectored vaccine gave protective immunity in NHPs at 1 year postimmunization, whereas a DNA vaccine did not [11]. Importantly, of the vaccines described here, only the PIV was the same as that in the clinical evaluation.

In phase I trials, all of the vaccine candidates were safe in the small cohorts tested and induced neutralizing antibodies to varying levels. Passive transfer of the sera of vaccinees to mice provided protection following ZIKV challenge, supporting the premise that neutralizing antibodies are likely a correlate of protection [6–8]. It is impossible to determine whether one vaccine candidate is superior because the published phase I studies focused on safety and used different neutralization assays; however, neutralization titers for each of the respective vaccine candidates were similar to those observed in preclinical studies. Overall, while huge progress has been made in Zika vaccine development over a period of 3 years, we still do not have a licensed vaccine nor a stockpiled candidate. This is in part due to limited clinical disease prevalence since mid-2016 when the virus ‘disappeared’, a feature of zoonotic viruses that come and go for unknown ecological reasons.

## Conclusions and future directions

Advancing platform technologies for potential vaccines as well as new virus vectors and expression systems offer enormous potential to generate candidate vaccines for emerging diseases at short notice and are foundational to the establishment of the Coalition for Epidemic Preparedness Innovations. This is exemplified by progress toward developing efficacious candidate vaccines against ZIKV. Given the limited knowledge of ZIKV when the epidemic started, these efforts provide optimism for improved rapid vaccine development against an emerging disease X. Notably, current vaccine efforts focus on emergency preparedness (i.e., stockpiling vaccines that give at least short-term protective immunity for 1 year) and not on routine immunization, requiring a vaccine that provides long-term protective immunity. Nonetheless, a major challenge of these efforts for emerging diseases is that they take place during acute outbreak scenarios and annual outbreaks are usually not observed. By the time a lead candidate vaccine has been developed, the outbreak has usually finished and we await future outbreaks to evaluate its efficacy. This is the situation with Zika virus, and we await phase II efficacy trials with candidate Zika vaccines when the next outbreak takes place. Finally, although surveillance efforts are critical in predicting when outbreaks will take place, it is not possible to undertake continued surveillance for all potential emerging zoonotic pathogens. Instead, platform technologies must be optimized for rapid responses to disease X at short notice.

## Abbreviations

DEN: Dengue virus
E: Envelope protein
JE: Japanese encephalitis virus
LAV: Live attenuated vaccine
NCR: Noncoding regions
NGS: Next generation sequencing
NHP: Nonhuman primate
NS: Nonstructural protein
PIV: Purified inactivated vaccine
prM: Precursor of membrane protein
YF: Yellow fever virus
ZIKV: Zika virus
